# Stelara struck: a case of noninfectious pneumonitis secondary to ustekinumab

**DOI:** 10.1186/s12890-022-02066-z

**Published:** 2022-07-19

**Authors:** Katherine A. Despotes, Christine L. Vigeland

**Affiliations:** 1 Department of Medicine, University of North Carolina, Chapel Hill, NC USA; 2 Marsico Lung Institute, University of North Carolina, Chapel Hill, NC USA; 3 Chapel Hill, USA

**Keywords:** Drug induced ILD, Ustekinumab, ARDS

## Abstract

**Background:**

We describe a case of acute hypoxic respiratory failure due to drug induced lung disease secondary to ustekinumab, which is a monoclonal antibody used to treat psoriasis, psoriatic arthritis, and inflammatory bowel disease.

**Case presentation:**

A 33-year-old man with a history of Crohn’s disease presented with fevers, myalgias, and abdominal pain, and subsequently developed acute hypoxemic respiratory failure approximately 2 weeks after restarting ustekinumab for his Crohn’s disease. Cross-sectional chest imaging showed ground glass opacities and bilateral consolidations. Due to progressive hypoxia, he ultimately required intubation and mechanical ventilation. Broad infectious and autoimmune work up was negative, making drug induced interstitial lung disease (DILD) the leading consideration. He was treated with high dose steroids with dramatic improvement in his respiratory status. At follow up, his imaging findings had largely resolved, and his pulmonary function tests were normal.

**Conclusions:**

For patients presenting with acute hypoxic respiratory failure, it is critical to identify the underlying cause. In addition to testing for common respiratory infections that can cause respiratory failure, patients should also be evaluated for risk factors for developing atypical or opportunistic infections as well as inflammatory pneumonitis. Due to receiving ustekinumab, our patient was both at risk for developing an opportunistic infection as well as DILD. Although rare, DILD is a recognized toxicity of ustekinumab. Ustekinumab can cause significant lung injury, as in our patient, but with steroids and avoidance of future doses of the medication, our patient demonstrated good recovery. Reassuring outcomes have similarly been described in the literature; however, this case provides further details about outcomes with long-term follow-up clinical, imaging, and pulmonary function testing data available. We recommend consideration of high dose steroids for these patients for whom DILD is suspected.

## Background

One of the most common diagnoses for admission to the intensive care unit (ICU) is acute respiratory distress syndrome (ARDS). ARDS is a syndrome characterized by sudden onset of hypoxic respiratory failure, with bilateral infiltrates found on chest imaging [1]. ARDS can develop as a result of both direct and indirect injury to the lung, and early identification of the underlying etiology is critical in order to provide directed therapy. The most common causes of ARDS are infectious, such as influenza, SARS-COV2, bacterial pneumonia, and sepsis [2]. However, non-infectious pneumonitis can also present as ARDS. We present a case of a patient who presented with ARDS likely secondary to ustekinumab, a biologic agent approved to treat psoriasis, psoriatic arthritis, and inflammatory bowel disease that blocks IL-12 and IL-23 [3].

## Case presentation

The patient is a 33-year-old man with history of Crohn’s disease who was admitted to the medical ICU with acute hypoxemic respiratory failure. He was in his usual state of health until 36 h prior to presentation, when he developed myalgias, fatigue, headache, and epigastric pain. He denied change in stools consistent with prior exacerbations of Crohn’s disease. Due to his symptoms, he was admitted to the hospital for further evaluation. His past medical history was notable for Crohn’s disease diagnosed in 2000, for which he had been on multiple immunosuppressants. Most recently he was trialed on ustekinumab (Stelara) 2 years prior to this hospitalization, with good response to therapy. However, he discontinued the medication after 5 months due to concerns about potential infectious complications. Two weeks prior to presentation, ustekinumab was restarted due to evidence of active Crohn’s disease. Importantly, prior to this hospitalization he had no respiratory symptoms and no evidence of interstitial lung disease found on prior CT scans.

**Fig. 1:**
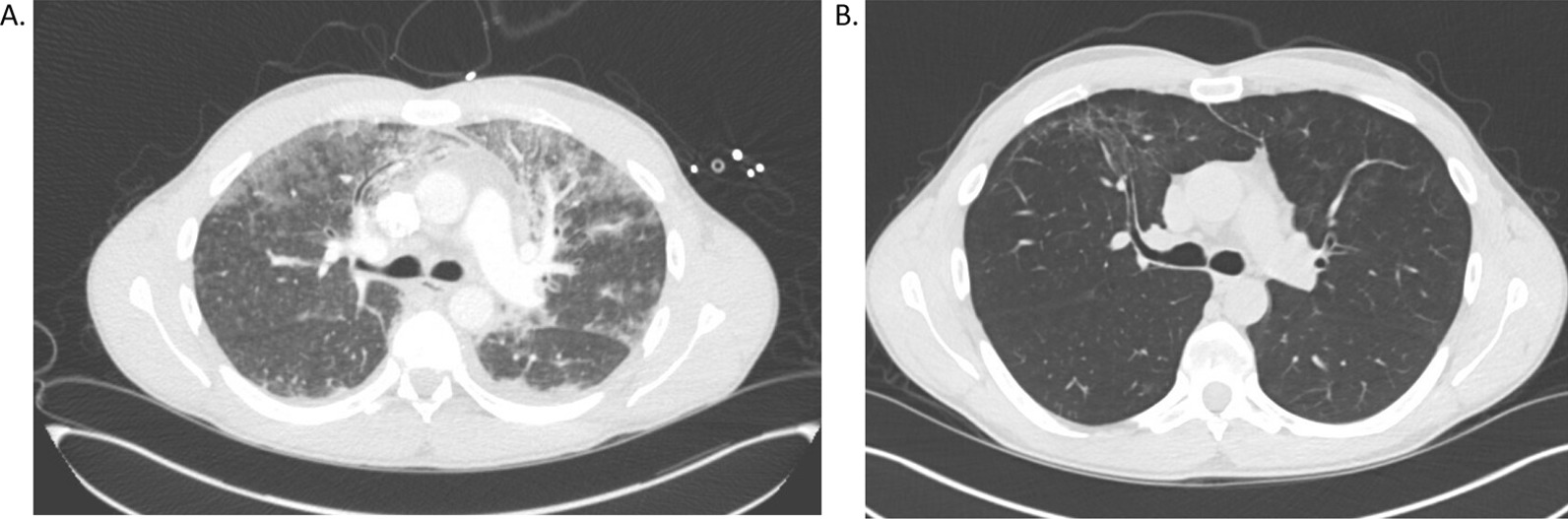
Representative CT images from **A** CTA chest obtained during initial hospitalization, and **B** follow up high-resolution CT scan (HRCT) obtained 6 weeks after hospital discharge

On presentation to the hospital, his vital signs were noteworthy for temperature of 39.6C, heart rate 120 beats per minute, respiratory rate 18 breaths per minute, blood pressure 117/83 mmHg, oxygen saturation (SpO2) of 98% on room air. Exam was remarkable only for epigastric tenderness on abdominal exam. His lungs were clear to auscultation bilaterally. Initial chest X-Ray was clear with no acute airspace disease. On hospital day 1, he developed cough, dyspnea, and hypoxemia, requiring 5 L nasal cannula oxygen to maintain SpO2 > 90%. His hypoxemia rapidly worsened, and on hospital day 2 he was moved to the ICU for initiation of high flow nasal cannula with 75% fraction of inspired oxygen (FiO2). Repeat chest X-Ray showed diffuse reticular infiltrates. At this time, our differential was infectious (bacterial, viral, or atypical pneumonia) versus inflammatory (Crohn’s disease associated pneumonitis, drug induced pneumonitis). Due to the concern for infectious etiology, he was intubated and a bronchoscopy was performed. Empiric antibiotic coverage with vancomycin, cefepime, and azithromycin was also started. CT chest with contrast showed bilateral consolidations and ground glass opacities without evidence of pulmonary embolism (Fig. 1A).

Laboratory work up was significant for an initial white blood cell count of 16.3 with 92% neutrophils and C-reactive protein (CRP) of 296.8 mg/L. Infectious workup was negative including blood cultures and respiratory pathogen panel. Bronchoscopy was performed that was also negative for bacterial, viral, and mycobacterial pathogens (Table 1). Serologic workup for underlying connective tissue disease-associated interstitial lung disease (ILD) was also negative (Table 2).

**Table 1:** Summary of infectious workup

Respiratory pathogen panel w/SARSCOV2 PCR	Negative
Blood cultures	Negative
Lower respiratory culture	Negative
Fungal antibodies	Negative
BAL bacterial culture	No organisms seen
BAL legionella culture	Negative
BAL AFB culture	No acid fast bacilli seen
BAL fungitell	< 31 pg/mL
BAL pneumocystis smear	Not detected
Urine histoplasma antigen	Negative

**Table 2:** Summary of serologic workup for underlying connective tissue disease

ANA	Negative
dsDNA	Negative
ENA	Negative
ANCA	Negative
ANCA‐MPO	Negative
ANCA‐PR3	Negative
Myositis panel	Negative
Rheumatoid factor	< 8.6 IU/mL
CCP antibodies	Negative

Given his negative infectious workup, his respiratory failure was thought to be most consistent with drug-induced pneumonitis versus Crohn’s-disease related ILD. He was treated with pulse steroids (methylprednisolone 1 g daily for 3 days) before being transitioned to prednisone 1 mg/kg/day (80 mg). Prednisone was tapered by 20 mg every 4 days until 20 mg daily, with plans to continue this dose until outpatient follow up. With steroid treatment, his respiratory status significantly improved. He was extubated on hospital day 9 and was discharged on hospital day 18. At the time of discharge, he was not requiring supplemental oxygen. The patient was seen in pulmonary clinic for follow up 6 weeks after hospital discharge to continue to monitor his clinical course and recovery. At that time, he had tapered his prednisone to 5 mg daily. Pulmonary function tests revealed normal forced vital capacity (FVC) and diffusion capacity of lung for carbon monoxide (DLCO). Repeat CT scan showed dramatic improvement in previously seen ground glass infiltrates (Fig. 1B). Given negative infectious and autoimmune work up, and dramatic improvement following cessation of ustekinumab, we favor his clinical case to be consistent with drug-induced interstitial lung disease (DILD) secondary to ustekinumab. He has continued to have normal lung function in the 12 months following his initial hospitalization, and no recurrence of respiratory symptoms, reassuring against fibrotic complications and arguing against other underlying ILD.

## Discussions and conclusions

This case highlights the challenge of diagnosing the underlying etiology of ARDS, particularly in patients with multiple potential etiologies. We considered a variety of infectious causes in this immunosuppressed gentleman, the potential for Crohn’s disease-associated ILD, and possible DILD related to ustekinumab, a rare but reported phenomenon. DILD is likely under recognized but thought to account for 3–5% of ILD cases in some registries, and is a diagnosis of exclusion with no specific or pathognomonic findings [4]. After careful exclusion of infectious etiologies, and now with the benefit of clinical follow up after cessation of ustekinumab, we think that DILD secondary to ustekinumab is the most likely explanation for this patient’s presentation.

The literature describing pulmonary toxicity related to ustekinumab is limited, but includes several case reports as well as one case series. The case series by Brinker et al. identified 12 patients who were prescribed ustekinumab for psoriasis that developed acute or subacute respiratory symptoms within 2 years of drug initiation [5]. All 12 cases involved need for hospitalization or medical intervention. The pulmonary manifestations described in these patients included interstitial pneumonia (7 patients), organizing pneumonia (1 patient), eosinophilic pneumonia (3 patients), and hypersensitivity pneumonitis (1 patient) based on a combination of imaging, BAL findings, and/or lung biopsy [5]. Another case report by Kalra describes a patient with Crohn’s disease who developed dry cough and dyspnea that progressed between the first and second doses of ustekinumab, and was subsequently diagnosed with chronic eosinophilic pneumonia based on imaging findings and BAL with 67% eosinophils [6].

Treatment in reported cases has largely consisted of discontinuation of ustekinumab, with or without steroids. In the case series by Brinker, 5 patients received steroids, 2 patients received antibiotics, 1 patient received cough suppressants, while six patients were treated with discontinuation of medication alone [5]. Ustekinumab has a long half-life, and therefore in addition to avoidance of further doses, the addition of steroids has been used to potentially hasten recovery [6]. As in our case, in the case report by Kalra, medication cessation and a prolonged steroid taper led to resolution of symptoms and imaging findings [6].

There are a few notable aspects of our patient’s case that are worth highlighting in comparison to other descriptions in the literature. Our patient had prior exposure to ustekinumab 2 years prior to presentation and only developed respiratory symptoms after the medication was reintroduced. This was a slightly longer period of time than what had been evaluated in the largest case series by Brinker, which excluded cases in which respiratory symptoms developed more than 2 years after ustekinumab initiation [5]. Another case report did describe a case of interstitial pneumonia that developed in a patient who had been treated with ustekinumab for 2 years [7]. Thus, close follow-up to evaluate for adverse reactions is essential, especially if there may be a temporal delay between medication initiation and adverse reaction.

The other notable aspects of this patient’s presentation were how rapidly his respiratory failure progressed and the severity of lung injury compared to most cases described. Only one patient in the Brinker case series required mechanical ventilation [5]. Fortunately, our patient did very well after initiation of steroids and avoidance of further ustekinumab. The majority of described cases have reported similarly favorable outcomes.

The expansion of ustekinumab approval for the treatment of inflammatory bowel disease in 2016 will likely lead to wider use of this medication, making awareness of the potentially serious complication of DILD increasingly important [3]. Conditions like Crohn’s as well as systemic lupus erythematosus (for which ustekinumab has been used off-label) can have associated lung disease, and therefore caution is warranted if considering ustekinumab in these patients. We report this case to increase this awareness among providers (including rheumatologists, gastroenterologists, dermatologists, and pulmonologists), and to provide guidance in management from our experience. Providers who suspect DILD from ustekinumab should consider high-dose steroids early on, once infection has been thoroughly evaluated, with taper as described over subsequent weeks, and avoidance of further ustekinumab dosing. The ustekinumab prescribing information was updated in 2018 to reflect the risk of lung inflammation with its use, and close monitoring with thorough investigation of new respiratory symptoms after initiation of this medication is warranted [3].

## Data Availability

Not applicable.

## Abbreviations

ICU: Intensive Care Unit
ARDS: Acute respiratory distress syndrome
CRP: C-reactive protein
DLCO: Diffusion capacity of lung for carbon monoxide
DILD: Drug-induced interstitial lung disease
FVC: Forced vital capacity
FiO2: Fraction of inspired oxygen
ILD: Interstitial lung disease
SpO2: Oxygen saturation
